# An accurate and adaptable photogrammetric approach for estimating the mass and body condition of pinnipeds using an unmanned aerial system

**DOI:** 10.1371/journal.pone.0187465

**Published:** 2017-11-29

**Authors:** Douglas J. Krause, Jefferson T. Hinke, Wayne L. Perryman, Michael E. Goebel, Donald J. LeRoi

**Affiliations:** 1 Antarctic Ecosystem Research Division, Southwest Fisheries Science Center, National Marine Fisheries Service, National Oceanic and Atmospheric Administration, La Jolla, California, United States of America; 2 Marine Mammal and Turtle Division, Southwest Fisheries Science Center, National Marine Fisheries Service, National Oceanic and Atmospheric Administration, La Jolla, California, United States of America; 3 Aerial Imaging Solutions, LLC, Old Lyme, Connecticut, United States of America; University of Siena, ITALY

## Abstract

Measurements of body size and mass are fundamental to pinniped population management and research. Manual measurements tend to be accurate but are invasive and logistically challenging to obtain. Ground-based photogrammetric techniques are less invasive, but inherent limitations make them impractical for many field applications. The recent proliferation of unmanned aerial systems (UAS) in wildlife monitoring has provided a promising new platform for the photogrammetry of free-ranging pinnipeds. Leopard seals (*Hydrurga leptonyx*) are an apex predator in coastal Antarctica whose body condition could be a valuable indicator of ecosystem health. We aerially surveyed leopard seals of known body size and mass to test the precision and accuracy of photogrammetry from a small UAS. Flights were conducted in January and February of 2013 and 2014 and 50 photogrammetric samples were obtained from 15 unrestrained seals. UAS-derived measurements of standard length were accurate to within 2.01 ± 1.06%, and paired comparisons with ground measurements were statistically indistinguishable. An allometric linear mixed effects model predicted leopard seal mass within 19.40 kg (4.4% error for a 440 kg seal). Photogrammetric measurements from a single, vertical image obtained using UAS provide a noninvasive approach for estimating the mass and body condition of pinnipeds that may be widely applicable.

## Introduction

Obtaining measurements of body size and mass is fundamental to pinniped research and population management. Simple metrics such as body length and mass provide valuable information about the age [1–4], physiology [5, 6], foraging ecology [7–10], life history, and evolution [11–14] of marine mammals. Importantly, the mass and body condition of marine predators can be an indicator of prey availability and habitat quality for managed populations [15–21], or serve as indices of ecosystem health [22–24]. Accordingly, there is a long history of attempts to estimate the size of free-ranging pinnipeds with measured success.

For studies with limited sample requirements, accessible pinnipeds may be captured and measured directly. Such hands-on measurements are typically accurate and are referred to as “manual” measurements. If it is impractical to weigh an animal due to its size or position, reliable mass estimates can be derived using multiple girth and blubber thickness measurements [25, 26]. Alternatively, mass can be estimated using only length and girth [27–31], which reduces capture times. However, the accuracy of such models varies widely, particularly for large (>400 kg) pinnipeds [28, 30, 31]. Moreover, manual techniques for collecting morphological data necessarily involve physical captures, which are time- and resource-intensive disturbance events that carry substantial risk for both animals and researchers. Finally, large pinnipeds are typically immobilized chemically which requires accurate mass estimates to properly administer powerful sedatives. Improper dosing can result in animal mortality [32, 33] and manual techniques do not provide *a priori* mass estimates. Experienced field personnel can often visually estimate mass within pharmaceutical safety margins. However, for problematic (e.g., [31]) or endangered species a more accurate, non-invasive approach may be needed.

Wildlife photogrammetry has offered opportunities to measure, *inter alia*, body size and nutritive condition from a distance (e.g., [34, 35]), thereby reducing animal disturbance, research effort, and risk. However, substantial challenges remain before photogrammetric approaches become practical in a pinniped management context. First, useful photographs must capture enough allometric information in a two-dimensional (2D) image to estimate mass. Second, photogrammetric methods must be scalable beyond a few individuals to be of use. Finally, experiments must be designed that address the inherent challenges of sampling wild pinnipeds. For many practical reasons, previous photogrammetric studies have circumvented these limitations by gathering data from anesthetized or trained animals. Consequently, the resultant size or mass-estimation models are often sensitive to animal movement or body alignment relative to the camera.

The recent proliferation of unmanned aerial systems (UAS) has provided promising new tools for wildlife monitoring and research [36–38]. When compared with manned aircraft, UAS are logistically simple, low cost, and safe [39–41]. Battery powered vertical takeoff and landing (VTOL) UAS, in particular, are well suited for collecting photogrammetric data from pinnipeds. Without need for a landing strip, they can operate in rugged environments, be launched quickly, and fly below cloud cover [42]. Furthermore, they are quiet, which limits disturbance to wildlife, and they are self-leveling, which improves photograph quality and simplifies image post-processing [43, 44].

Historically, leopard seals (*Hydrurga leptonyx*) have been difficult to sample because they are typically solitary [45], associated with marginal pack ice habitat [46–49], and are particularly cryptic in their distribution [50, 51]. However, some leopard seals congregate seasonally in higher densities near mesopredator colonies [52–57]. The population of leopard seals at Cape Shirreff, Livingston Island, Antarctica is comprised of seasonally resident, marked individuals [58] and, therefore, presents a unique opportunity to test the performance of a VTOL UAS photogrammetry approach.

Here, we assess a non-invasive method for determining leopard seal size, condition, and mass, based on vertical images taken from VTOL UAS using individuals of known body size. Aerial photogrammetric and manual measurements are compared to: 1) test the accuracy of pinniped body measurements obtained using aerial photogrammetry, and 2) compare the precisions of both manual and photogrammetric measurements. We subsequently examine the sensitivity of photogrammetric measurement accuracy to changes in the haul-out substrate, body position of target animals, and the altitude of the UAS. 3) We build and evaluate several modeling approaches to estimate the mass of this large pinniped using straight-line distances measured from a single 2D photograph. Finally, we discuss the utility of a body-condition index for leopard seals and other phocids.

## Materials and methods

### Study site

Field studies were conducted at Cape Shirreff (62.47°S, 60.77°W) on the north shore of Livingston Island, Antarctic Peninsula. This field site was selected because it provided access to a seasonally resident population of leopard seals that regularly haul-out along the coast [57, 58]. Photographic missions over leopard seals, conducted in conjunction with ongoing monitoring studies [59], were completed in January and February of 2013 and 2014.

### UAS platform

**Fig 1:**
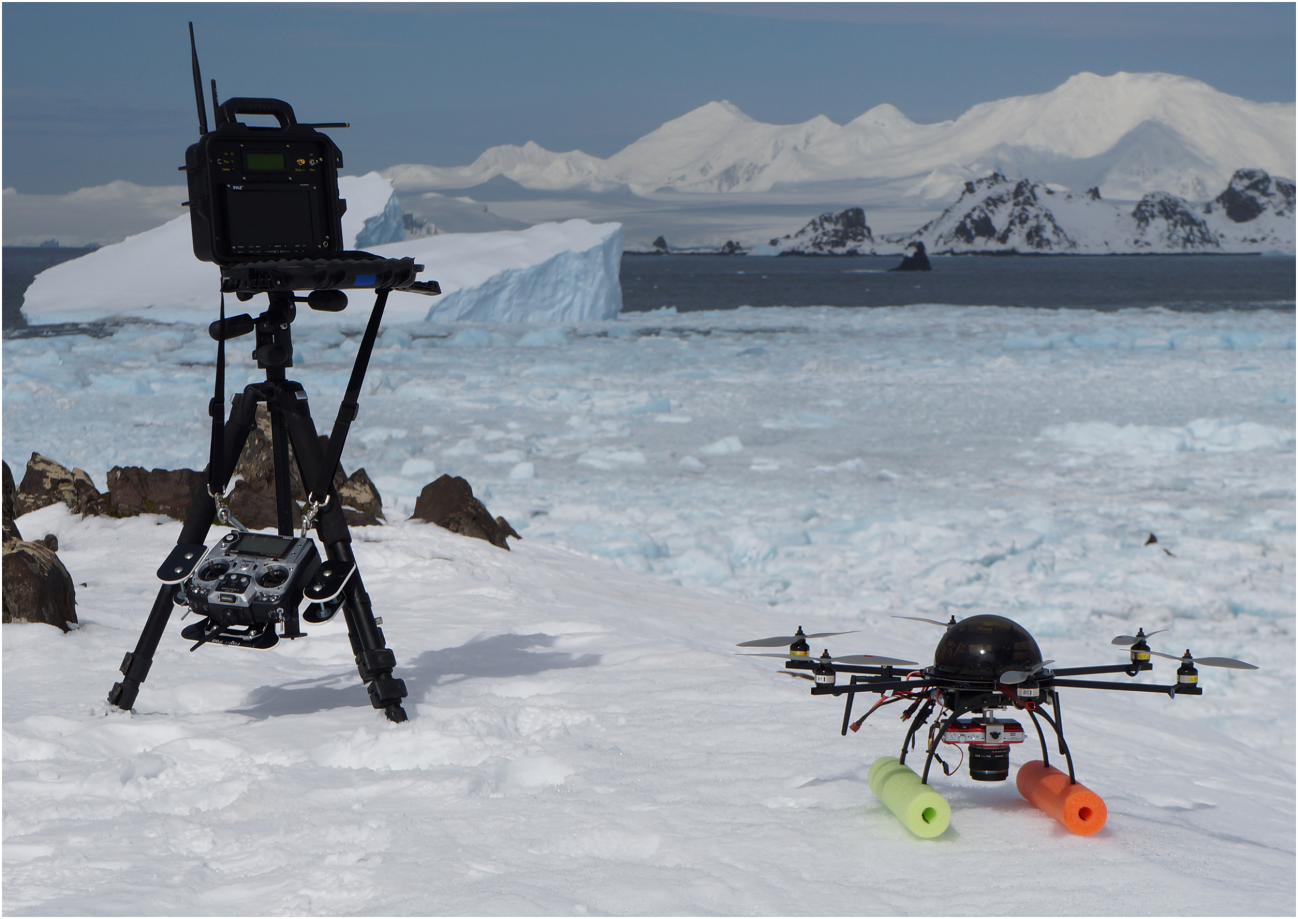
The APH-22 VTOL UAS system. Photo Credit: D. Krause/NOAA.

The APH-22 (Aerial Imaging Solutions, Old Lyme, CT) is a battery powered VTOL UAS system which was described in previous studies [43, 44]. It consists of a 2.4GHz radio transmitter, and weatherproof hexacopter and ground station with a live video display (Fig 1). It was chosen for its portability, durability, high-resolution photography, and its stability in flight across a variety of weather conditions [43] despite its low weight (1.2 kg, payload capacity: 1 kg). Our field configuration featured a downward facing Olympus E-PM2 digital camera (16.1 Megapixel, Micro Four-Thirds format, 0.23 kg) with an Olympus M.Zuiko 45 mm f/1.8 lens, and a single battery (QuadroPower 6200 mAh Li-PO, 0.13 kg) as payload. The camera was set to record Large Super Fine JPEG and RAW images, ISO 1250, aspect ratio: 4:3, Shutter Priority Mode (shutter speed 1/2000). The 45 mm lens was designed for the E-PM2 Micro Four-Thirds sensor, allowing undistorted coverage across the entire photograph. Calibration flights utilizing a medium contrast (8:1) resolution target (RST-704, Series C) produced undistorted photographs with a ground-resolved distance of 1.0 cm at 30 m altitude (Perryman unpublished data).

### Leopard seal capture protocol

Healthy adult female leopard seals were selected and chemically immobilized [57, 60]. While sedated, the following manual morphometrics were taken to the nearest 0.5 cm from seals in ventral recumbancy (prone position): standard length (SL), curviliniear length (CL), and axillary girth (AG) [61]; an additional umbilicus girth (UG) was taken at the mid-point posterior to the rib cage and anterior to the hip girdle. SL was taken using a measuring tape affixed to a rigid pole; a weighted plumb line was used to increase accuracy (Table 1). In 2014, SL was measured by three independent observers (readings were not shared) to estimate observer variance. Girths and curvilinear lengths were measured with a calibrated line. Each animal was weighed to obtain mass (M) using a sling, tripod, hand winch, and a tensionometer (MSI-7300 Dyna-Link 2, capacity 1,000 ± 0.5 kg).

**Table 1:** Photogrammetric (P) and manual (M) measurements used in regression models.

Measurement	Measurement Type	Abbreviation
Standard Length	M	SL
Curvilinear Length	M	CL
Axillary Girth	M	AG
Umbilical Girth	M	UG
Standard Length	P	PSL
Overall Length	P	POL
Curvilinear Length	P	PCL
Axillary Width	P	PAW
Umbilicus Width	P	PUW
Width 1	P	PW1
Width 2	P	PW2
Width 3	P	PW3
Width 4	P	PW4
Width 5	P	PW5
Width 6	P	PW6
Width 7	P	PW7
Width 8	P	PW8
Width 9	P	PW9
Width 10	P	PW10

The units for all straight-line measurements were cm, mass was recorded in kg.

After manual measurements were taken, sedative-reversal pharmaceuticals were administered [60]. Each animal’s recovery was visually monitored until it reached a mobile state. After handling, all animals in this study were re-sighted at least once within two weeks of capture in a healthy state.

### UAS flight protocol

One aerial survey flight was conducted over each leopard seal immediately following capture (n = 14), or within 24 hours (n = 3), to reduce measurement error between measured and estimated mass. The rapid sedative-reversal recovery times (2.68 ± 2.08 min) ensured that coverage was obtained from non-sedated, free-ranging leopard seals in multiple, natural body positions.

Before capture events the APH-22 system was set up and calibrated >50 m from the target animal. After the animal recovered from the procedure, missions were flown to target elevation before approach to decrease potential disturbance [62]. Two reference markers, either 6 or 10 m apart, were placed near target animals to provide a known-distance scale reference. Aerial photographs were taken every 2 seconds above target seals from altitudes of 23, 30 and 45 m. The number of photographs taken per seal ranged from 6 to 74.

### Data analysis

**Fig 2:**
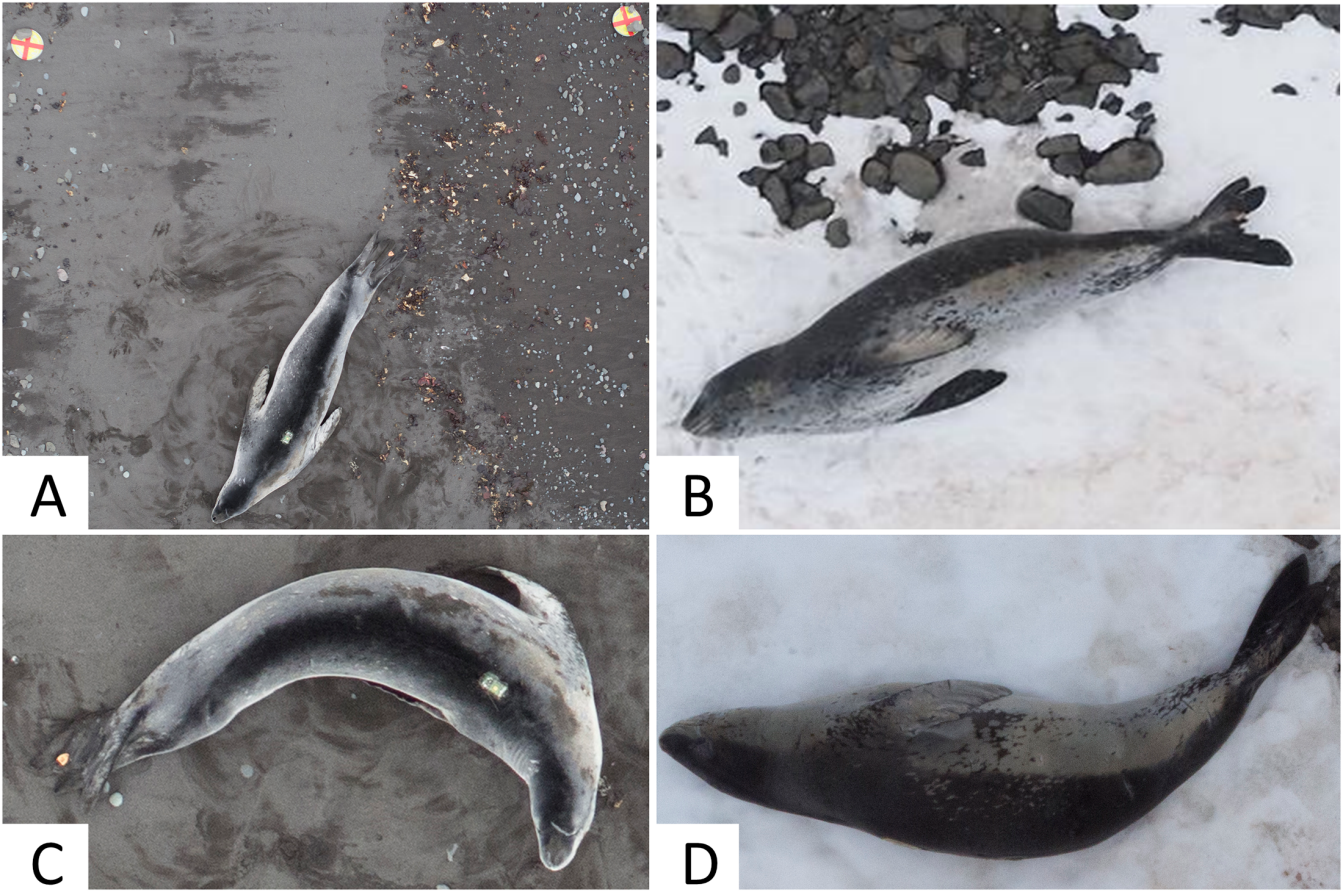
Example photos of leopard seal body positions and substrates. A) A dorsal-straight body position on sand substrate. The ground reference scale is marked by two red crosses. B) A lateral-straight body position, C) a dorsal-curved body position, and D) a lateral-curved body position.

Representative photographs containing the entire leopard seal and a ground scale reference were selected from each target altitude (23, 30, 45 m). For each photo, the substrate under the seal (snow or sand) and two categorical variables of seal body position (POS1: straight or curved; POS2: dorsal or lateral) were recorded (e.g., Fig 2). Sample sizes per category can be seen in Table 2. Usable photos were obtained from 100% of target animals (n = 17), and 76% of target animals (n = 13) provided images from either multiple altitudes, multiple body positions or both. All references to the seal’s identity were removed, and photographs were measured by three independent observers to assess measurement variation.

**Table 2:** Measurement accuracy comparisons.

Test Category	Treatment Group	n	Group Mean Error ($\overset{-}{X}$ ± *sd*)	Df	F	p
UAS Altitude	45 m	23	1.96 ± 0.92%	47	0.047	0.954
	30 m	13	2.07 ± 1.49%			
	23 m	14	2.03 ± 0.87%			
Body Position 1	Dorsal	40	2.06 ± 1.08%	48	0.067	0.879
	Lateral	10	1.81 ± 1.00%			
Body Position 2	Straight	31	2.15 ± 1.08%	48	1.468	0.222
	Curved	19	1.77 ± 1.01%			
Substrate	Snow	38	2.14 ± 1.13%	48	1.06	0.385
	Sand	12	1.61 ± 0.70%			

Four unbalanced one-way ANOVA test results which compared measurement error (“group mean error”, calculated as % Error) between PSL and SL. For each ANOVA photographs (n = 50) were grouped according to test category. The null hypotheses that mean error was not different between treatment groups could not be rejected for any test category. Similarly, no differences were detected between UAS altitude treatment groups using a Tukey’s HSD test: 45m-30m (p = 0.955); 30m-23m (p = 0.978); 45m-23m (p = 0.996).

**Fig 3:**
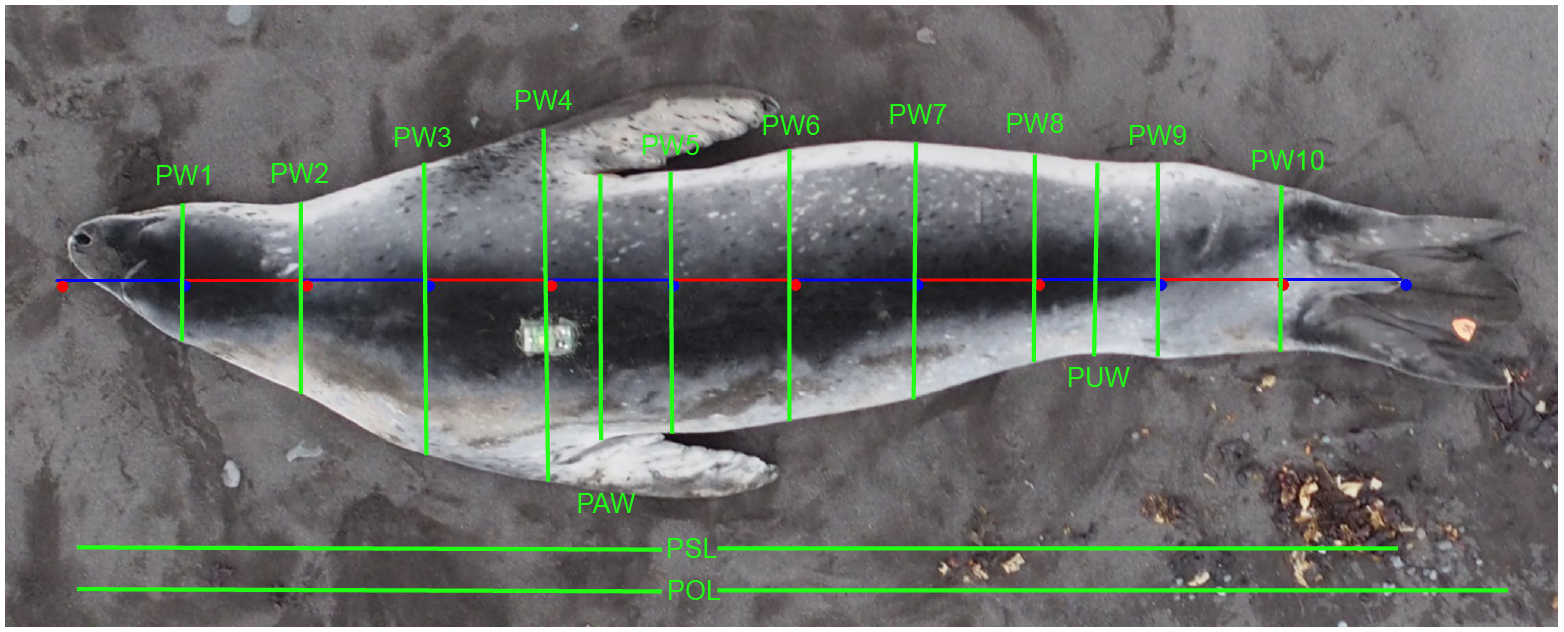
Photogrammetric measurements. An example measured leopard seal with labeled photogrammetric measurements.

Manual measurements are denoted by identifiers like SL for standard length and photographic measurements are denoted with a P prefix (e.g., PSL for photographic standard length) (Table 1, Fig 3). Images were measured in pixels (*Pixels*) using ImageJ, a Java-based open access software package [63]. A customized Java script (S1 Fig) allows for semi-automated photo processing. The user defines markers (nose to tail) on the image and the script creates 10 equidistant width landmarks. The photogrammetric measurements were standard length (PSL), overall length (POL), widths (edge to edge of the animal measured orthogonal to PSL) at landmarks 1 through 10 (PW1-PW10), axillary width connecting the bases of the fore flippers (PAW), and umbilicus width at the midpoint between the anterior rib cage and hip girdle (PUW).

Subsequent data analysis was conducted using R 3.1.1 (R-Core-Team 2015). A straight line distance on an object can be calculated exactly from a photograph as long as the lens focal length and the distance from lens to object are known [64, 65]. The focal length (*fl*) of our lens was 45 mm, and the pixel width (*pw*) was calculated from the camera sensor size and the sensor resolution [66]. Photograph pixel measurements were converted to ground distance by converting pixel measurements to photo distance: *Photo Distance* = *Pixels* × *pw*. Then, ground distance was calculated using the altitude of the UAS as the distance from sensor to object [67]:

$$Ground\;Distance=\frac{Altitude}{fl}\times Photo\;Distance$$ (1)

Three data sets were created for further analysis: 1) the “accuracy” data set, which contained all photogrammetric measurements (n = 50) from 17 animals at up to 3 altitudes, 2) the “manual” data set of all ground-based measurements from each seal that was both captured and aerially surveyed (n = 17), and 3) the “mass-estimation” data set, which contained one set of photogrammetric measurements for each aerially surveyed seal (n = 17). Standard parametric assumptions of ordinary least squares regressions (independence, normality, linearity and homoscedasticity) were verified for each data set [68, 69]. The photographs from two individual seals were stitched from multiple images. That process and resultant pixilation stretch resulted in outlying measurements which were removed from the manual and mass-estimation data sets (final sample size, n = 15).

### Precision tests

We addressed three precision-related questions: 1) is the photogrammetric protocol clear enough that naïve observers consistently derive similar measurements? We compared the precision of our photogrammetric observers using measurements of POL. 2) Is the variance between replicated measurements of the same seal by different observers equal to or lower than traditional (ground) methods? 3) On average, did the ground and photogrammetric observers obtain similar measurements? For questions 2 and 3 we compared a straight-line measurement of standard length that was common to both manual (SL) and photogrammetric (PSL) techniques.

First, POL measurements were compared among observers in order to assess individual measurement variance. Comparisons were made using balanced one-way ANOVA tests among observers, and Tukey’s Honest Significant Difference (HSD) test between paired observers. Then, we assessed the equivalence between the variances of ground versus UAS-derived measurements using a Levene’s test to compare the residuals (a sum of the absolute differences between each observer’s estimate and the mean for a given animal) for SL (n = 9) and PSL (n = 9). Finally, the SL and PSL means were compared with a two-group, independent, paired t-test [70].

### Accuracy tests

For comparisons of accuracy, manual morphometrics were assumed to be “true”. For PSL, and SL measures from 2014, the three-observer mean value was used. Measurement error was calculated as the percent difference between mean PSL and mean SL as follows:

$$\%\;Error=|(1-\frac{SL}{PSL})\times 100|$$ (2)

Subsequently, potential changes in photogrammetric measurement accuracy (% Error) due to differences in substrate, seal body position, and UAS altitude were tested using unbalanced one-way ANOVA and HSD tests.

### Mass estimation

Leopard seal body mass (M) was estimated using ordinary least squares (OLS) linear and power regression, and linear mixed effects models. Multiple linear regression models were evaluated for multicollinearity using the variance inflation factor (VIF) test [68] and all candidate models were excluded due to values of √VIF > 2. For each set of models, M was the dependent variable and all photogrammetrically-derived measurements were potential predictor variables. All subset combinations were run for each family of regression analysis [70, 71]. The most informative models were identified within each family using Akaike’s Information Criterion (AIC) [72], and selected between families by minimizing residual standard error.

Each photogrammetric measurement was recorded by each of three observers; however, OLS models require single measurement values per animal. Therefore, linear and power regression models were evaluated for each observer separately as well as using multiple-observer mean values. To account for the possibility that multiple-observer mean values may affect model outputs by masking measurement error variation, we analyzed the relationship between observer and predicted mass using a linear mixed effects model [73, 74]. Photogrammetric measurements were scaled to unit variance and included as fixed effects for each observer and animal. Observer was considered a random effect with random intercepts and slopes [75].

Previously published leopard seal mass-estimation models [28, 31] were also run using ground-based leopard seal measurements to evaluate their performance. The level of significance used for all tests was *P* < 0.05. All values are listed as mean ($\overset{-}{X}$) ± standard deviation (*sd*) unless otherwise indicated.

Leopard seal interactions and captures were conducted in accordance with Marine Mammal Protection Act Permit No. 16472 granted by the Office of Protected Resources, National Marine Fisheries Service, and the NMFS-SWFSC Institutional Animal Care and Use Committee Permit No. SWPI2011-02. Cape Shirreff has been designated as an Antarctic Specially Protected Area by the Commission for the Conservation of Antarctic Marine Living Resources. Access to the study site was granted in accordance with Antarctic Conservation Act Permit No. 2012–005.

## Results

Twenty-two UAS surveys were flown on 15 days, providing coverage of 15 individual leopard seals including 50 sample images at a variety of altitudes, substrates, and body positions. Flights were conducted in a variety of weather conditions including snow (n = 1) and fog (n = 2), but most flight days were partly-cloudy across a spectrum of light conditions. The mean wind speed was 7.22 ± 4.85 (range: 1–18) knots, and the mean flight duration was 7.37 ± 3.14 min. We did not observe any behavioral responses to the UAS from leopard seals when the altitude was ≥ 23 m.

### Precision tests

**Fig 4:**
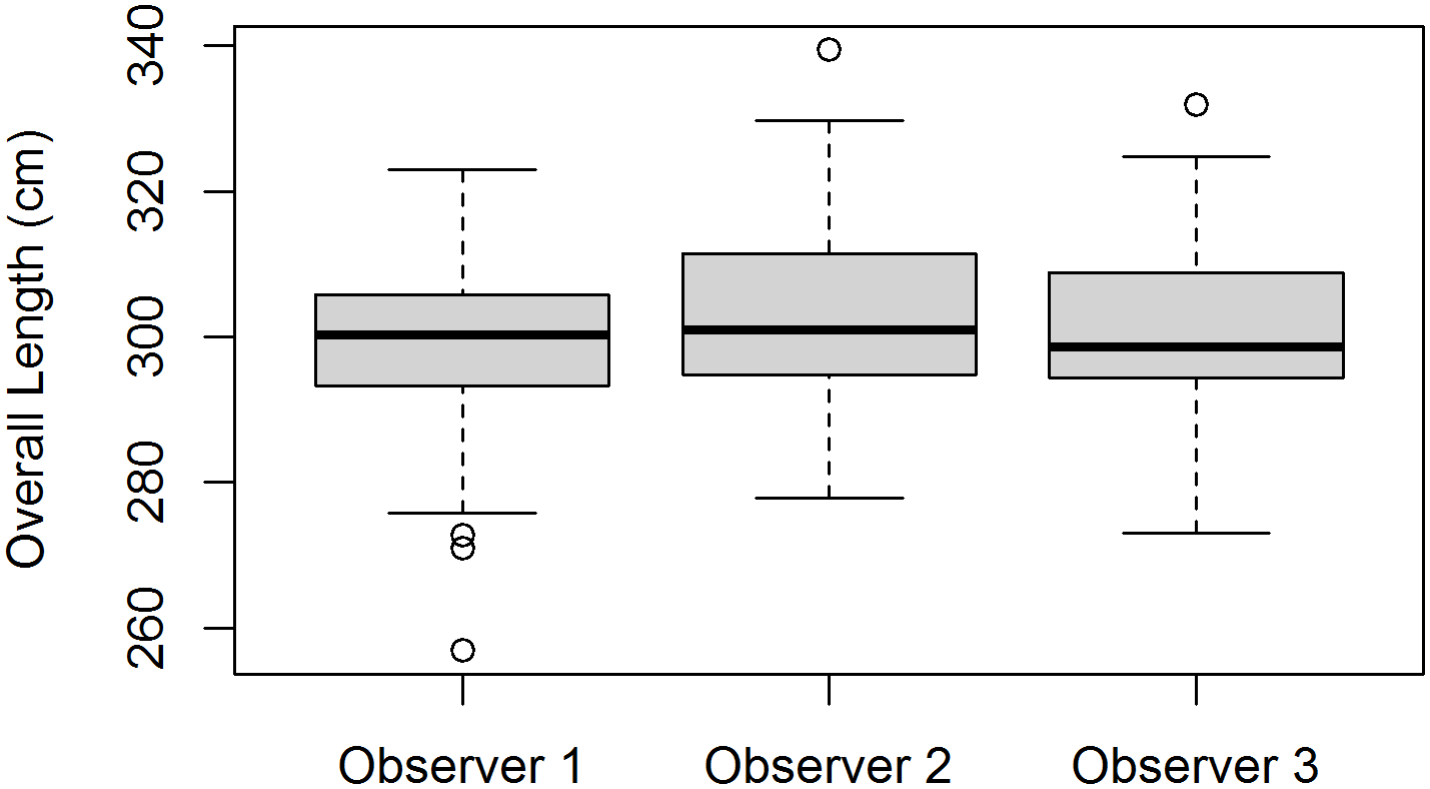
Observer precision. Photogrammetrically-derived measurements of leopard seal overall length (n = 50) compared between three independent observers (ANOVA F_2,147_ = 2.009, p = 0.138).

**Fig 5:**
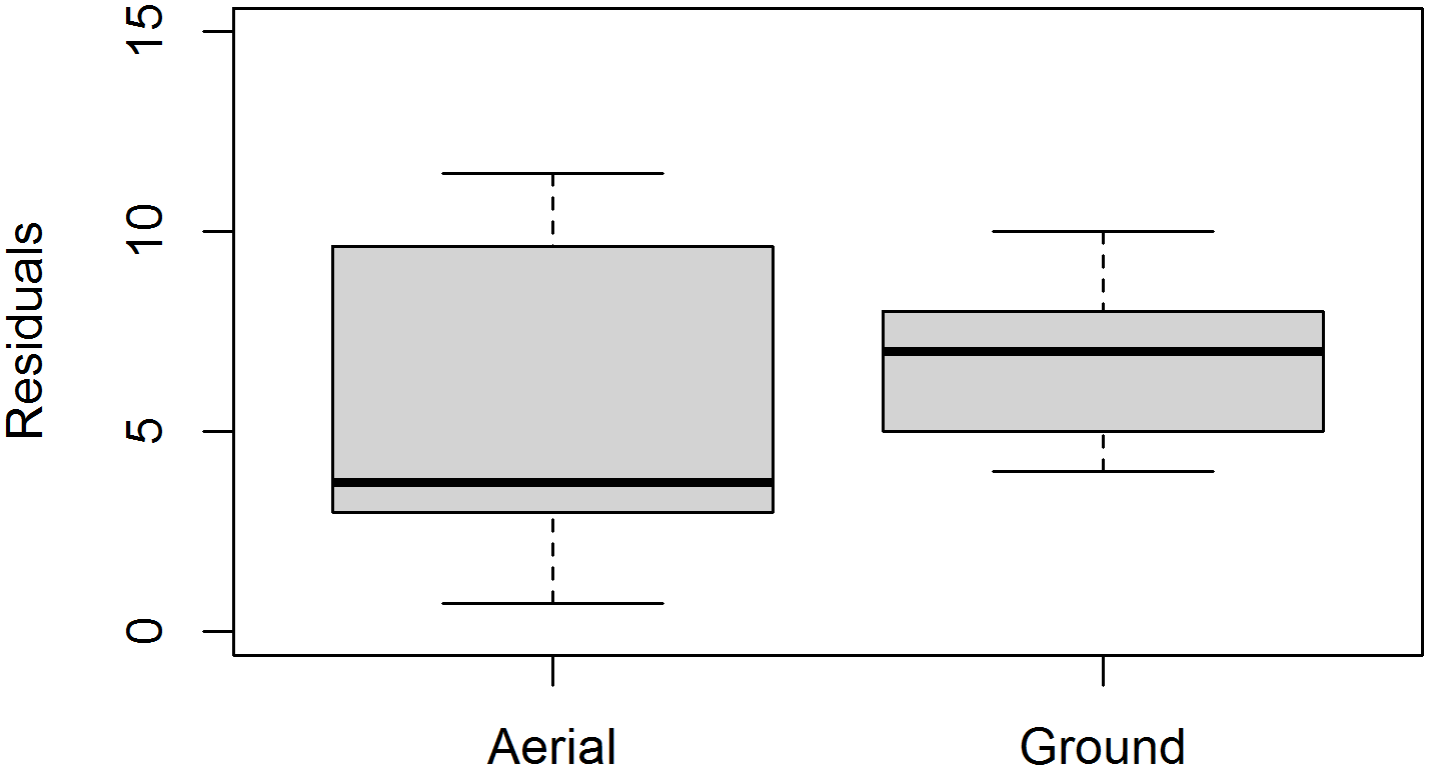
Aerial versus ground precision. Observer-derived variances compared between ground-based SL (n = 9) and aerial PSL (n = 9) measurements. Residuals were calculated by summing the absolute difference between each measurement and the mean value for a given animal. Leven’s Test F_1,9_ = 2.439, p = 0.14.

No significant differences were detected for POL among observers (Fig 4, n = 50, ANOVA F_2,147_ = 2.009, p = 0.138; Tukey’s HSD: Obs2 –Obs1 p = 0.1307, Obs3 –Obs1 p = 0.852, Obs3 –Obs2 p = 0.343). The observer variance associated with UAS-derived measurements of PSL did not differ significantly from ground-based measurements of SL (Fig 5, F_1,9_ = 2.439, p = 0.14).

Mean values of SL (301.30 ± 1.73 cm (*se*), n = 9) and PSL (302.75 ± 1.48 cm (*se*), n = 9) obtained from multiple observers of the same seal from the same day were not significantly different (t_9_ = 1.0767, p = 0.3096, $\overset{-}{X}$ of the differences = 1.427 cm).

### Accuracy tests

The mean % Error of PSL for all photographs in the study (n = 50) was 2.01 ± 1.06%. PSL was highly correlated with corresponding manual measurements (r = 0.85, p << 0.001). No differences in PSL % Error were identified among photographs grouped by UAS altitude, substrate, or body position (Table 2).

### Mass estimation

The most informative mass estimation model equations and their corresponding AIC, R^2^ values, and residual standard errors are listed in Table 3 for each family of models. Results based on previous, manual measurement approaches are also listed for comparison. All listed models featured significant mass predictive ability; however, the photogrammetrically-derived models had substantially lower residual error. While both manual and photogrammetric model evaluations selected for a measure of seal length (SL or POL), umbilicus width (PUW) outperformed girth as a predictor of leopard seal mass.

**Table 3:** Comparison table of the most informative model from each model family.

Model Family	Reference	Equation/Formulation	Adjusted R^2^	P	AIC	Residual Standard Error (kg)
Linear mixed effects	This Study	M = POL × PUW + (1+POL × PUW | Observer)	--	<< 0.001	406.6	± 19.40
Linear regression	This Study	M = 8.21 + 0.028(POL × PUW)	0.870	<< 0.001	85.115	± 16.04
Power regression	This Study	log(M) = -0.73 + 0.576 × log(POL × PUW^2^)	0.887	<< 0.001	-123.273	± 28.47
Linear regression	Hofman 1975	M = 1.31(SL × G^2^/2.83 × 10^4^)	0.307	0.019	110.165	± 36.98
Power regression	Van den Hoff et al. 2005	log(M) = 0.774 + 0.921 × log(SL × G^2^)	0.394	0.007	108.921	± 48.58

The linear mixed effects model shows the model formulation as per R package lme4 (Bates et al. 2015) (n = 45), while regression models are described with equations (n = 15). The residual standard error for power regression models was back-transformed from log space.

**Fig 6:**
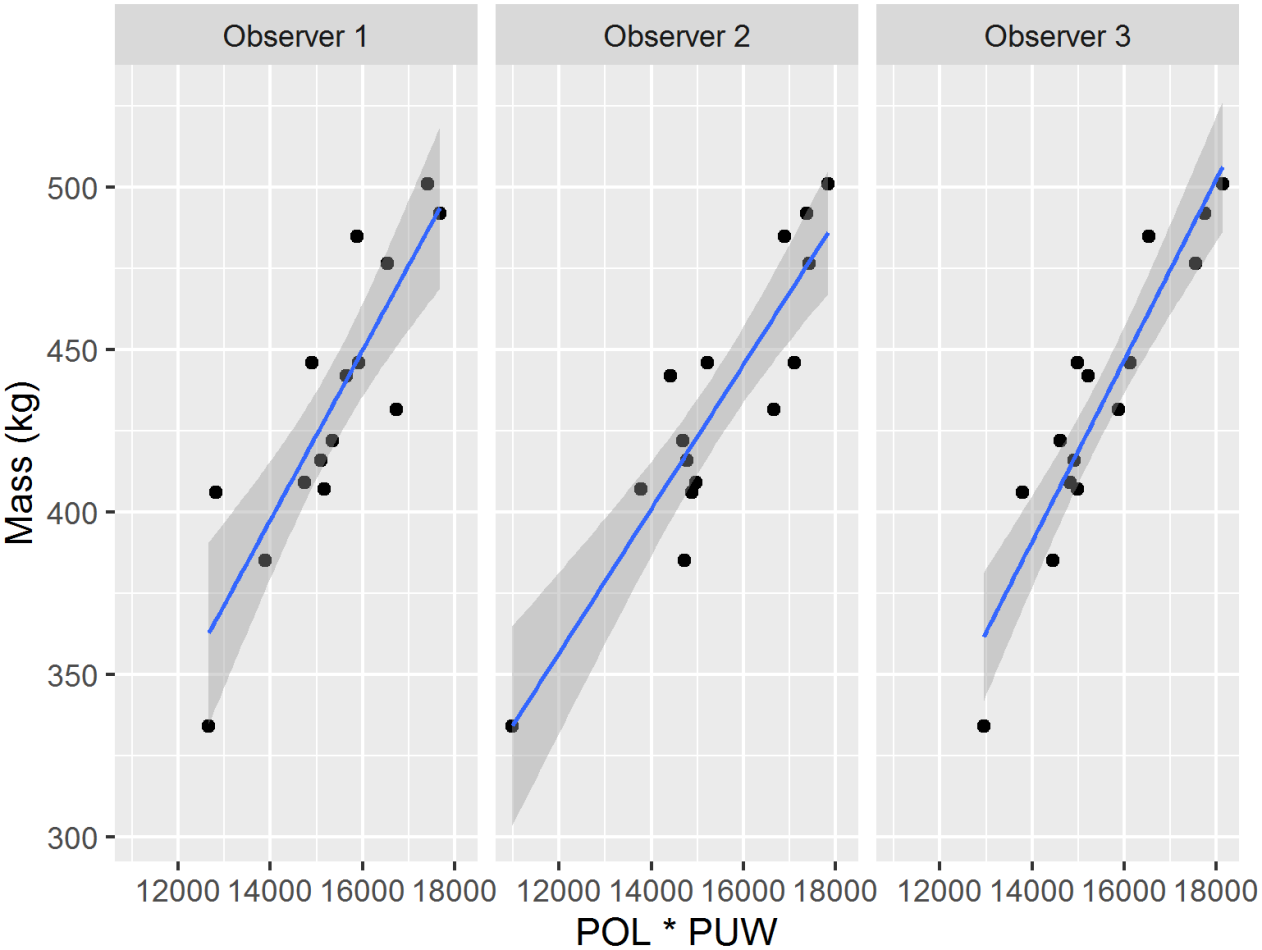
Linear mass estimation models per observer. Linear regressions of overall length (POL) times umbilicus width (PUW) to leopard seal mass with 95% confidence intervals (gray ribbon) for each observer. For all models n = 15, R^2^ > 0.85, and P << 0.0001.

Inter-observer measurement variance was low across precision tests and linear regression models per observer were extremely similar (Fig 6). Moreover, the leading linear regression model using multi-observer mean values had the lowest standard residual error (± 16.04 kg, or 3.6% error for a 440 kg leopard seal). However, explicitly considering observer-based measurement variance within a linear mixed effects model resulted in an increased residual standard error of ± 19.40 kg (or 4.4% error for a 440 kg seal).

## Discussion

### Precision and accuracy

Two-dimensional photographs provided highly-accurate mass estimates for adult leopard seals based on body measurements from vertical overhead images. The straight line measurement accuracy in this study (~2%) was as, or more, accurate than previous pinniped photogrammetry reports. Further, leopard seals are large pinnipeds [76], which has been an historically challenging size class for mass-estimation models, and their streamlined body shape [31] contains elements of both phocid (e.g., spindle-shaped body) and otariid (e.g., long neck, developed fore-flippers) dimensions. Therefore, a successful photogrammetric technique for leopard seals may be applicable to other pinnipeds. Historically, comparative studies found manual measurement models were more accurate than photogrammetric models [77–79]. To our knowledge, this is the first report in which a photogrammetric model was more accurate (Table 3). Such accuracy probably resulted from a combination of limited measurement error for each model predictor and the added body-width information from overhead photographs.

In general, photogrammetry should facilitate high measurement precision and low inter-observer measurement variance (e.g., [80]). Unlike measurements taken in the field, each observer has an identical view of the target animal unencumbered by changing field conditions, movement, etc. And, photogrammetric software facilitates precise, repeatable measurements [63]. By comparison, information on the measurement precision of manual techniques is limited. In fact, Hofman [28] directed future research programs to conduct repeat manual length measures on captured pinnipeds to increase accuracy and estimate precision. With exceptions (e.g., [78, 79]), however, most studies have not reported variance for manual measurements. Our protocol resulted in low variance between independent observers (e.g., Fig 4), and photogrammetric measurement variance equivalent with the historical “gold standard” manual technique (Fig 5). The nearly identical standard error for PSL vs. SL and the fact that differences could not be detected between mean values emphasize the precision of this approach.

Additionally, the high precision and accuracy within this study population was consistent irrespective of body position or substrate. The use of sedation-reversal pharmaceuticals facilitated rapid recovery from immobilization (~2.5 min). Hence, manual measurements were conducted on sedated leopard seals, yet proximate UAS flights captured images of mobile animals in multiple body positions. Subsequent comparisons of the measurement error between various flight altitudes, substrates, and an array of natural leopard seal body positions did not detect significant differences between any groups.

### Historical estimates of mass and volume

Mass is inherently related to body volume. Logically, many previous mass-estimation studies, both manual and photogrammetric, attempted to estimate the volume of individuals and relate it to mass. Such approaches have produced some promising but variable results (Table 4). Attempting to estimate animal volume creates myriad challenges related to deriving three dimensional (3D) models from 2D photographs, including obtaining sufficient measurements in field settings, accounting for substrate and body position which can effect volume estimates, and correcting for changes in body composition which can alter seal density and volume independently (e.g., [81]).

**Table 4:** A summary of pinniped mass estimation studies.

Category		Reference	Species	Key Measurements	N	Mass-Estimation Error ^A^
Morphometric		Hofman 1975	LS,WS,RS,CES	Length, Girth^2^	31	± 8.1%
		Kooyman and Castellini 1990	WS	Length, Girth^2^	12	Not Reported ^B^
		Castellini and Calkins 1993	SSL	Length, Girth^2^	390	± 0.98%
		Van den Hoff et al. 2005	LS	Length, Girth^2^	51	± ~34% ^C^
Photogrammetric	Single Camera	Hayley et al. 1991	NES	Side Area, Girth Area, Length	70	± 12%
		Bell et al. 1997	SES	Girth Area, Side Area	45	± 4.71% ^D^
		McFadden et al. 2006	MS	Girth Perimeter, Lateral Perimeter	26	Not Reported ^E^
		Ireland et al. 2006	WS	Overhead Width, Side Area, Side Height	73	± 13.8%
		Meise et al. 2014	GSL	Adjusted Length, Girth	♂ 15	± 7.46% ^F^
					♀ 21	± 13.54% ^G^
		This Study	LS	Overall Length, Umbillicus Width	15	± 4.4%
	Stereo	Waite et al. 2007	SSL	Length, Girth^2^	53	± 9.0%
		de Bruyn et al. 2009	SES	Volume	13–40	± 0.57–8.54%

^A^ “Mass Estimation Error” represents the lowest error from a given study derived either by cross validation, or residual standard error. Species: leopard seal (LS), Weddell seal (*Leptonychotes weddellii*, WS), Ross seal (*Ommatophoca rossii*, RS), crabeater seal (*Lobodon carcinophaga*, CES), Steller sea lion (*Eumetopias jabutus*, SSL), northern elephant seal (*Mirounga angustirostris*, NES), southern elephant seal (*Mirounga leonina*, SES), Hawaiian monk seal (*Monachus schauinslandi*, MS), Galapagos sea lion (*Zalophus wollebaeki*, GSL).

^B^ R^2^ = 0.87.

^C^ based on the reported back-transformed error of 120 kg for an approximately 350 kg seal.

^D^ error reported as 95% confidence intervals (CI).

^E^ error was reported as a 95% CI of ±5 kg, but mean true mass not reported; therefore the range of potential error is extremely large.

^F^ based on reported errors for a 75 kg male.

^G^ based on reported errors for a 65 kg female.

Three dimensional (3D) volumetric models of pinnipeds have been created to estimate mass using multiple photographs from multiple angles [82, 83]. While promising for limited (e.g., underwater) applications [82], they require either complex, synchronized, multi-camera set-ups, or are overly sensitive to animal movement [83]; however, recently developed post-processing corrections may improve error rates (R. Beltran, pers comm.). Another approach involves taking 1 or 2 photos (e.g., lateral, anterior, posterior) of a seal at ground level from a known distance and regressing the photo-derived surface area to approximate volume [77, 78, 84]. Though reasonably accurate, this approach was only recommended [78] for sedated animals or specific groups of pinnipeds, like northern elephant seal (*Mirounga angustirostris*) bulls or Hawaiian monk seals (*Monachus schauinslandi*) [84], that haul-out alone and tolerate close approach by humans. Pinniped approach distances can be increased by supplementing manual scale references with an accurate estimate of camera to seal distance [67]. For example, Meise et al. [79] utilized a laser distance meter with their single camera system. However, their measurements (Table 4) were sensitive to body position, and changes of substrate. Despite being less invasive than hands-on approaches, all of these techniques have caveats that make them impractical for many field applications. Lateral photo techniques do not work well for crowded haul-outs where neighbors obscure target animals. It would be difficult to scale these techniques up to cover a large population and all of them involve working in proximity to target animals.

### Less error more information

Improving manual morphometric or photogrammetric approaches is an exercise in maximizing information per observation and reducing measurement error. There is error in all measurements, and those errors can be compounded or masked when not properly specified within models [75, 85]. Therefore, ideal approaches should reduce error in data acquisition and data processing, and employ models that take known variance into account.

Utilizing a single vertical image from a VTOL UAS facilitated a suite of features that reduce error in data acquisition including: a vertical photo angle that obviates complex lateral angle distortion corrections [67, 79, 86], a lens matched to the camera sensor, which reduces image distortion [44], and the inherent slow speed and low-altitude of the UAS supports high-resolution photography. While it is possible to obtain overhead images of seals from the ground, the process is cumbersome [87]. Our single-camera, single-photo approach provided more accurate and reliable (robust to changes in altitude, substrate and body position) estimates of mass than previous studies which used multiple images. Because there is a given amount of measurement error introduced per photograph it follows that limiting the number of necessary photographs limits error.

Altitude was calculated from ground scale markers in this study, but such markers are not necessary for two reasons: First, the high accuracy of the APH-22 Freescale MPX4115A air pressure altimeter produces a ground measurement error of < 1% [44], and second, laser altimeters are now common which can further increase altitude accuracy by measuring the absolute lens to target distance. Also, many previous approaches require multiple photos of the same animal or multiple-photo lens calibrations, but each additional image increases user effort and the potential for error. Therefore, a single photograph, single camera approach inherently limits measurement error.

Estimates of pinniped mass are also subject to errors derived from non-proportional changes in body measurements. Phocids, in particular, experience large changes in percent body fat as they fast during the breeding season (e.g., [81, 88]), and subsequently forage (e.g., [89]). Further, body composition estimated from girths may introduce error because differences can be due to changes to muscle or fat [90]. And, whereas mass changes with percent body fat, many morphometric characteristics do not [91]. For example, adult pinniped length does not change with mass [92] or any other factor [1], and changes in other measurements may be masked by rigid skeletal structures (e.g., skull, thorax, and hip girdle). Therefore, measurements from non-skeletally-restricted body regions are more likely to inform changes in body condition [35]. Our results are consistent with this view. For example, PUW was more correlated with leopard seal mass than any other measurement and it was an important predictor in all photogrammetric mass-estimation models (Table 3).

Error assessments for field studies focused on measuring large, free-ranging mammals face inherent challenges. For example, sample sizes are typically low and, as we illustrated, ground-truth measurements may have variances similar to or greater than the comparison group. Therefore, we suggest that studies focused on measuring body size in wildlife populations should explicitly consider observer bias when possible. Using a single observer or averaging across observers may artificially reduce error in model predictions, and moreover, such results should not be applied broadly. Using multiple observers permits the explicit assessment of measurement variance, and the correction of any systematic measurement error. And, identifying observer as a random effect illustrates the strong performance of our model irrespective of observer. Further development of image-assessment automation may also reduce human observer bias.

### Allometric measurement selection

The difficulties of volume estimation can be limited by deriving targeted mass-estimation models from easily-defined, straight-line body measurements. Demonstrated allometric scalings between body parts and traits, such as mass, are pervasive in the natural world [13, 14, 93]. However, allometric measurement errors often change with body size. Approaches and measurements that work well for a given body size may lose efficacy for larger size classes (e.g., [30]). Therefore, an approach that provides flexibility to quickly develop models focused on an animal group of interest can limit measurement error.

The leopard seals at Cape Shirreff during January and February are actively foraging adult females [58], presumably recovering body fat following their breeding season. In the absence of established body part to mass allometric relationships, we collected multiple, regularly-spaced widths (Fig 3) from overhead photos. This broad approach facilitated the identification of several informative allometric measurements, including PUW. And, within this limited size class our measurement error varied constantly with body size. Further testing is required to determine if PUW is equally useful for other size classes of leopard seals or other pinniped species. But, it is likely that width measurements from non-restricted body regions will strongly correlate with mass because those dimensions change more directly with fluctuations in percent fat.

### Body condition

Tracking the responses of pinnipeds to environmental changes will continue to be vital to understanding and managing marine ecosystems. Leopard seals are a particularly important apex predator in Antarctic coastal systems that affect mesopredator (e.g., penguin, Antarctic fur seal (*Arctocephalus gazella*)) populations [58, 94–96] and consume the ecologically-important Antarctic krill (*Euphausia superba*) [97, 98]. While focal studies of demographics, diet, and foraging behavior are needed, simple indices of predator body condition may be valuable indicators of ecosystem health. A practical index should reflect body condition and be collected over appropriate scales. While mass estimates are biologically valuable, they do not provide information on the nutritive state of an animal (e.g., a long seal could have a high mass compared to a short seal even while starving). The fineness ratio (length/maximum diameter) has been used to study the swimming efficiency of fish and pinnipeds [99, 100], and a modified version (SL/height) was suggested as a condition index for leopard seals [31]. Although useful for small-scale monitoring, height must be obtained from the ground, which is not viable for sampling over large areas.

In comparison to ground techniques, however, unmanned aerial approaches can easily be scaled to larger regions. For example, including gear preparation and transportation each leopard seal capture in this study took 4.2 ± 0.6 hours and involved a crew of five people. Conversely, UAS flights (including packing, transportation, set up, calibration, and flight time) which provided equivalent body condition data took 0.37 ± 0.08 hours with a crew of two. In combination with coastal vessels, mesoscale (200–600 km) UAS surveys of marine mammals have been conducted in a few weeks [101, 102]. In situations where manned aircraft are impractical UAS provide an inexpensive and scalable pinniped survey platform.

We suggest that monitoring programs expand the use of UAS platforms, and use non-skeletally-restricted width measurements to develop body condition indices for pinnipeds. Because PUW correlates more strongly with mass change for the leopard seals than maximum diameter (R^2^ = .78 and R^2^ = .38 respectively), an appropriate condition index (CI) would be:

$$CI=\frac{POL}{PUW}$$ (3)

Another major advantage of an index is that relative measurements such as these can be obtained from any aerial platform with no need for absolute scale.

## Conclusions

Aerial photographs derived from a VTOL UAS and processed by amateur, volunteer observers using open source software provided precise and accurate estimates of body size and mass for large (>400 kg), free-ranging, adult leopard seals. Our approach both limits observer error and specifically accounts for it in resulting mass estimates. Results were robust to changes in substrate and body position. While our linear mixed effects model works well for this population, its performance for other size classes or species remains unknown. However, models can be developed easily with appropriate data, and our approach collects accurate allometric data that likely will be successful in other systems. Our sampling approach is promising for focal or longitudinal monitoring studies of leopard seals, and perhaps other pinnipeds, without the need for costly, invasive animal captures [92]. We believe similar approaches could be scaled to increase spatial coverage and provide body condition indices for ecosystem-based resource management. We suggest expanding future studies to integrate larger sample sizes and new pinniped species to verify and quantify the efficacy of UAS photogrammetry.

## Supporting information

S1 Fig Semi-automated photogrammetric measurement routine. Java script written as a macro for Image J.
(PDF)
