# Supplementary material for: An accurate and adaptable photogrammetric approach for estimating the mass and body condition of pinnipeds using an unmanned aerial system
Source: PLoS One. 2017 Nov 29;12(11):e0187465. doi: 10.1371/journal.pone.0187465 (PMC5706667; doi:10.1371/journal.pone.0187465)
Supplement: S1 Fig — Java script written as a macro for Image J. (PDF) [file pone.0187465.s001.pdf]

```
// The macro "Ten Widths" color codes and draws dots
 // at 10 equally spaced points along a selected line.
 // Written by D.J. Krause, E. Keen and H. Fearnbach.

 macro "ten widths [k]" {
  getLine(x0,y0,x11,y11,width);
  dx = x11 - x0;
  dy = y11 - y0;
  xstep = dx/11;
  ystep = dy/11;

  // Interpolate segment pts
  x1 = x0 + 1*xstep;
  x2 = x0 + 2*xstep;
  x3 = x0 + 3*xstep;
  x4 = x0 + 4*xstep;
  x5 = x0 + 5*xstep;
  x6 = x0 + 6*xstep;
  x7 = x0 + 7*xstep;
  x8 = x0 + 8*xstep;
  x9 = x0 + 9*xstep;
  x10 = x0 + 10*xstep;

  y1 = y0 + 1*ystep;
  y2 = y0 + 2*ystep;
  y3 = y0 + 3*ystep;
  y4 = y0 + 4*ystep;
  y5 = y0 + 5*ystep;
  y6 = y0 + 6*ystep;
  y7 = y0 + 7*ystep;
  y8 = y0 + 8*ystep;
  y9 = y0 + 9*ystep;
  y10 = y0 + 10*ystep;

//Color code each line section
setTool("brush");
setColor("blue");
setLineWidth(4);
drawLine(x0,y0,x1,y1);
setColor("red");
drawLine(x1,y1,x2,y2);
setColor("blue");
drawLine(x2,y2,x3,y3);
setColor("red");
drawLine(x3,y3,x4,y4);
setColor("blue");
```

```
drawLine(x4,y4,x5,y5);
setColor("red");
drawLine(x5,y5,x6,y6);
setColor("blue");
drawLine(x6,y6,x7,y7);
setColor("red");
drawLine(x7,y7,x8,y8);
setColor("blue");
drawLine(x8,y8,x9,y9);
setColor("red");
drawLine(x9,y9,x10,y10);
setColor("blue");
drawLine(x10,y10,x11,y11);

//Color code each oval
setTool("brush");
setColor("blue");
setLineWidth(4);
fillOval(x0,y0,15,15);
setColor("red");
fillOval(x1,y1,15,15);
setColor("blue");
fillOval(x2,y2,15,15);
setColor("red");
fillOval(x3,y3,15,15);
setColor("blue");
fillOval(x4,y4,15,15);
setColor("red");
fillOval(x5,y5,15,15);
setColor("blue");
fillOval(x6,y6,15,15);
setColor("red");
fillOval(x7,y7,15,15);
setColor("blue");
fillOval(x8,y8,15,15);
setColor("red");
fillOval(x9,y9,15,15);
setColor("blue");
fillOval(x10,y10,15,15);
setColor("red");
fillOval(x11,y11,15,15);

  }

// Macro to save all measurements and data from a measured image.
// Saves JPEG with “draw” lines, and results table as .csv into the folder
```

```
// the image was selected from.
// Written by D.J. Krause

macro "photosave[f]" {

//store filename and datetime
filename = getInfo("image.filename");
getDateAndTime(year,month,dayOfWeek,dayOfMonth,hour,minute,second,msec);
newfilename = "IMGJ-Measured-"+filename;
tt = filename + "-Measured.csv"
mfile = getInfo("image.directory") + newfilename;
rfile = getInfo("image.directory") + tt
saveAs("Jpeg",mfile);
saveAs("Results",rfile);
print(year + "-" + month + "-" + dayOfMonth + " " +
hour + ":" + minute + ":" + second  +
"-- Measured leopard seal image and Results saved to" + mfile + "!");

}
```
